# Fostering patient-centredness by following patients outside the clinical setting: an interview study

**DOI:** 10.1186/s12909-020-1928-9

**Published:** 2020-01-15

**Authors:** Christel Grau Canét-Wittkampf, Charlotte Eijkelboom, Saskia Mol, Dorien Zwart, Iris Hendriks, Esther de Groot

**Affiliations:** 1 Julius Center for Health Sciences and Primary Care, University Medical Center Utrecht, University Utrecht, Utrecht, The Netherlands; 2 Wilhelmina Kinderziekenhuis, University Medical Center Utrecht, University Utrecht, Utrecht, The Netherlands

**Keywords:** Patient-centredness, Realist approach, Longitudinal integrated clerkship, Qualitative research, Interview study

## Abstract

**Background:**

Patient-centredness is considered a core competency for health professionals. To support faculty in designing courses focused on patient-centredness, an understanding of how educational interventions lead to patient-centredness is required. This study aims to show how learning mechanisms, which potentially contribute to patient-centredness, are triggered.

**Methods:**

Thirty-five third-year medical students at the UMC Utrecht followed four different patients for two years. The intervention took place in an out-of-hospital setting. Students visited patients in their home circumstances and accompanied them to clinical events. Twelve students were interviewed. The realist approach was used to construct configurations which relate components of the intervention to the context and learning mechanisms**.**

**Results:**

Following patients in their home circumstances for a prolonged period supported the development of meaningful relationships between students and patients and provided continuity. In the context of a meaningful relationship and continuity, mechanisms contributing to learning patient-centredness were triggered. The most important learning mechanisms found in this study were: reflecting, contextualising disease in a real persons’ life, broadening perspectives and engaging with the patients.

**Conclusions:**

Learning mechanisms are triggered by continuity and by meaningful student-patient relationships. These can be enhanced by an out-of-hospital setting and longitudinal contact. Thus, a relationship between students and patients is an important enabler for the development of patient-centredness.

## Background

There is an increasing emphasis on patient-centredness in medical education. Many different educational interventions with the aim to enhance patient-centred care have been designed and evaluated [1]. While there has been much attention to *whether* educational interventions lead to patient-centredness, little attention has been paid on *how* these interventions lead to patient-centredness. When developing an educational intervention, knowledge on how various aspects of an intervention lead to the intended outcome is required. Thus, to support faculty in designing interventions focused on patient-centredness, an understanding of how educational interventions lead to patient-centredness is necessary.

Patient-centredness is considered a core competency for health professionals [2]. Patient-centred healthcare is respectful of and responsive to individual patient preferences, needs and values. It ensures that patients’ values guide all clinical decisions [3]. The literature demonstrates that providing patient-centred care is associated with increased patient satisfaction, engagement, adherence to medical advice, and sometimes even improved health outcomes [4–8]. Various definitions of patient-centredness and models of patient-centredness exist [9, 10]. Scholl et al. identified and analysed distinctive dimensions of the concept of patient-centredness to develop an integrative model [8]. In this study, Scholl’s model was used to guide our data collection.

Health institutions aim to enhance patient-centredness by extending the duration of contact between patients and medical students. New models of longitudinal integrated clerkships (LICs) are increasing rapidly throughout the world [11–16]. A review discussing 58 studies has shown short-term successes of LICs. Students report having enhanced their patient-centred skills, having more empathy with patients, and they demonstrate a deeper understanding of the psychosocial component of the biopsychosocial model [11]. A follow-up study showed that LIC students held more patient-centred beliefs over time compared to rotation based clerkships [12]. Educational interventions where students follow patients in a non-clinical setting seem to foster patient-centredness as well [13, 14, 17–19]. However, all the studies above did not focus on *how* changes in a specific context as a result of an intervention contribute to patient-centredness.

To obtain insight into how an educational intervention leads to patient-centredness a realist approach is a sound choice. Realist studies focus on identifying underlying causal mechanisms by which an intervention works and exploring under which conditions they work [20, 21]. This is done by looking at CIMO (Context – Intervention - Mechanism - Outcome) configurations. These provide insight into how an intervention influences the context so that it triggers learning mechanisms which, in turn, lead to the outcome [15]. A realist review by de Groot et al. (in press, [16]) looked at educational interventions where patients play an active role. The authors identified learning mechanisms related to the development of caregivers’ patient-centredness. However, empirical knowledge on how different educational interventions to enhance patient-centredness change the context and what learning mechanisms are triggered remains scarce.

This study focusses on an adjusted form of a LIC program, which was introduced in the UMC Utrecht in 2015. In this educational program called the patient panel program, students followed patients in an out-of-hospital setting for two years [22]. The aim was to promote patient-centredness in students during their clinical development. The purpose of this qualitative research was to understand how learning mechanisms, which potentially contribute to patient-centredness, were triggered in medical students, who participate in an educational intervention where they meet patients in an out-of-hospital, longitudinal setting. Therefore, our research questions were:

- How does change in the learning context occur when students take part in a patient panel intervention?
- What learning mechanisms, which might contribute to patient-centredness, are triggered by changes in the context?

## Methods

### Intervention: patient panel program

A pilot of the patient panel program started in the third year of a six-year curriculum during the first clerkship in family medicine. Medical students followed patients for two years. Each student was coupled to four patients: a patient newly diagnosed with cancer, a chronic patient, a frail elderly and a pregnant woman or a new young family. Students followed patients both at home, in the hospital and at their general practitioners’ (GP) clinic. Students made scheduled visits to their patients twice a year, with the freedom to extend the number of visits. Students and patients were free to choose the location of the encounters. The focus in the intervention was on the interaction between student and patient. In contrast to contacts during hospital clerkships, students did not have time limits or time pressure when visiting their patients. When possible, students accompanied their patients to the hospital or other health care settings when there was a significant medical event. Each student was mentored by the patients’ GP. With their GP mentor, students could discuss their patients, the encounters, their own role and development, and other issues.

The students’ role in the intervention was described as ‘companion to the patient on their medical journey’. This non-clinical role was chosen to ensure student identification with the perspective of the patient rather than a medical doctor’s perspective. Besides, the literature suggests that following patients in a non-clinical setting enhances patient-centredness [13, 14, 17–19]. A feasibility study, done by Mol et al. at the end of year one of the patient panel pilot, contains a more detailed program description [16].

Ethical approval for this study was obtained by the Dutch Association for Medical Education (NVMO).

### Participants

Thirty-five students participated voluntarily in the pilot; seven of them did not participate in the second year of the pilot for different reasons, for example, study delay. Out of 28 students, seventeen students were invited by email for the interviews. Five out of the seventeen students declined study participation; one because of dissatisfaction with the patient panel, four students because of practical or personal issues. The other twelve students (eight women /four men) signed written informed consent and were interviewed in 2017, after having one and a half years of experience with the patient panel.

### Data collection and analysis

A research student (IH) performed semi-structured face-to-face interviews. The interview questions were based on the different components of patient-centredness described in the model of Scholl et al. (2014) (Additional file 1) [8]. The interview aimed to get a comprehensive idea of *how* students learn patient-centredness, and *what* triggers their learning process. Therefore, the interview questions gave room for students to describe the patients in their contexts, with their illnesses, how patients coped with their diseases and how they experienced their relationships with medical professionals.

Students also reflected on their relationship with the patients. Explicit questions about what the student learned in terms of patient-centredness were not asked with the intention to avoid as many socially desirable responses as possible. In the analysis of the interviews, we focused on the learning processes (according to the realist approach in our study).

All interviews were anonymised and transcribed. The first transcription was openly coded by two researchers (CGCW, CE) independent of each other, and supervised by an experienced researcher (EG). Three more transcriptions were coded by the two researchers (CGCW, CE) independently. Both researchers compared the coded fragments and discussed similarities and differences to refine the coding structure. The last eight transcriptions were each coded by one of the two researchers separately, and doubts were discussed. After the axial coding of all transcriptions, selective coding took place. Using constant comparisons, we constructed CIMO configurations to relate components of the intervention to context and learning mechanisms.

## Results

**Fig. 1:**
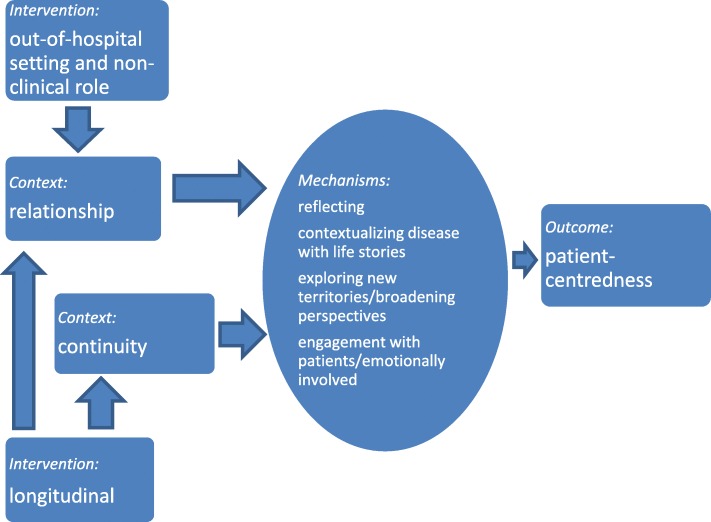
CIMO-configurations: the relationship between intervention, context, learning mechanisms and outcome in the patient panel program

The out-of-hospital setting and longitudinal aspect of the intervention stimulated continuity and meaningful student-patient relationships. In these contexts, learning mechanisms were triggered (Fig. 1).

### Relationship

Students met with patients outside the clinical setting, where they had a non-medical role. These components changed the relationship between students and patients, from a medical student-patient relationship that students were used to in a clinical setting, to a deeper and more meaningful student-patient relationship.

*Quote: It makes for a different kind of bond. You’re coming into someone’s home. ... So really you’re stepping into a private space. Then people [at home] are very different from how they are in the hospital. ... I think that when people are in their own world it gets a bit easier. Of course, they look at me differently, not just as a co-assistant at the hospital. You talk about other things as well, I think, when you invite someone into your home, you’re quite vulnerable. You show them all kinds of photos of outings, fun stuff like that, and you reveal a lot more of yourself then. (student 106).*

*Quote: Especially that you can give this patient more time to talk, and also talk about other things than you would with the patients in the residency. You really get into the physical things, and also some background on how to deal with things psychologically. You have all the space you need for this. So that bond is quite different. (student 115).*

*Quote: What’s the difference between these patients, for example, and the patients in your residency? R: It’s so different. I know these people as people. I know more about their personal life. It’s not a clinical relationship. (student 126).*

Students described that they gained more insight into patients’ daily life compared to a clinical setting. The meaningful relationship allowed students and patients to talk about profound matters related to the patients’ lives, (family) relationships, needs and beliefs. As a result, students were able to *contextualize disease with life stories.*

*Quote: I think I’ve achieved the goal, that at least you have a fuller picture of the patient, what’s happening to them and what their life looks like. And now I have two with MS, and the man with cancer is going through a very tough time. I don’t think it’s like that for everyone, because their life is really just their illness and that’s it. I do think I have a more complete picture of what that means for someone. (student 106).*

In getting to know their patients and their patients’ environment better, students *explored new territories and broadened their perspective.* As a result, students understood their patients’ point of view better.

*Quote: You learn to understand a bit, who someone is and what they think is important. But mainly how they combine their illness with their daily life and everything that is still on their mind except, let’s say, the medical aspects. (student 117).*

This out-of-hospital setting and context of a meaningful relationship triggered students to *reflect on what they experienced in the patient panel program*.

*Quote: … [F]or the patient I mentioned first, the one in her late twenties with lots of comorbidities, they decided to put her on artificial ventilation at home… The patient says that there are lots of conflicts between specialisms ... What actually frustrates her is that her oxygen saturation is always good, but she’s always short of breath and the doctors think that, well okay, if your saturation is good, you won’t be short of breath and that hurts her. ... It’s not so black and white. ... If she says she’s breathless, then that is how she feels and she wants help for it. Then you can discuss how exactly you should approach it medically, but in any case you should realise that it’s not so much about… I mean, if a patient feels something like that then it is true for that patient. And then you must do something about it. (student 123).*

Besides the out-of-hospital setting, also the longitudinal character of the intervention influenced the relationship. Students expressed a striking difference between the short-term relationship they were used to in a clinical setting and the longitudinal relationship in this intervention.

*Quote: We got to know each other a bit better because of that longer period. We developed a kind of trust relationship and that’s why he told me so much. (student 126).*

In a developing relationship, students got *emotionally involved and showed engagement* with their patients. Moreover, in time, patients trusted the students more and therefore told more about their perspective.

*Quote: I found it truly heartbreaking. He was having such a hard time with it, the fact that his daughter didn’t care about those photos at all. And he just sat with that box [of photos]. So I asked: would you like, shall we look at the photos together? And I spent a long time looking at photos with that man. (student 123).*

### Continuity

The longitudinal nature of the patient panel program provided continuity. Students talked about the difference between seeing patients in the hospital for just ten minutes and the continued contact they had with their patients in the patient panel program. By seeing the patient before, during and after medical appointments and by knowing about patients’ lives at home, students felt better informed about the context and perspective of the patient. This made them *contextualise disease with the lives of the patients:*

*Quote: [A]nd every time you see people looking forward to this appointment. Then after 10 min they’re back outside again and life goes on. Then here they come again, two weeks later, but for them, hidden behind those ten minutes in the doctor’s surgery lies something so much bigger: what does it do to them, the decisions the doctor makes at such a moment, what impact does it have on the rest, the rest of the week for those people? (student 129).*

*Quote: It’s like getting onto their life train for a moment and just riding along with them for a while. That’s why you don’t take a snapshot [of a particular moment], but continue over time. Sometimes you see a patient every three months in your surgery, but then I think the focus is on whether the medication is working, should the dosage be increased or reduced, so to speak. And now you really have a piece of their life, how is their whole life really doing? And you’re really experiencing it. (student 123).*

Students also described patient contacts in which learning was triggered less. This was not related to certain students; every student had more and less valuable patient contacts. It did seem to be related to the quality of the relationship with their patients. Less learning took place when students described a lack of connection with a patient or a negative relationship. In these cases, students described less context and perspectives of the patients. Students described several reasons for lack of connection, for instance: patients seemed to be uninterested in spending time with the student, sharing their story or involving the student in their lives, or a lack of personal connection or common ground.

*Quote: Like I said, I think it has a lot to do with feeling that the patient wants you [there]. With some patients I get the feeling that they’re just not hanging about waiting for me. They think it’s okay if I come by twice a year. We have our own life. I always feel with my patient, the pregnant woman, who’s had her child, that it’s okay if I’m with her for half an hour and then go away, because we’re busy with our own lives. (student 130).*

*Quote: Maybe it’s a personality clash. As well as factors of your own, like feeling tired yourself. These things play a role. I also think it’s mutual, it must come from both sides. When I talk to that woman with MS, she’s great and I can stay with her for an hour [no problem] but I think we both know that there is no click. It must come from both sides […] and you just have more of a click with some people than others. (student 106).*

## Discussion

This study explored medical students’ learning experiences, within a longitudinal program where students followed patients in an out-of-hospital setting. Our data suggest that following patients in their home environment for a prolonged period supports the development of meaningful relationships between students and patients. Furthermore, following patients for a prolonged time provided the possibility of continuity in the relationship with the patient. In the context of a meaningful relationship and of continuity learning mechanisms related to patient-centredness were triggered, such as contextualizing disease with life stories and exploring new territories [16].

Our analysis showed that the experience of a meaningful student-patient relationship was an enabler for the development of patient-centredness. Previous literature suggests that LICs are effective in enhancing patient-centredness due to the longitudinal design. This enables students to build relationships with their patients [11, 15, 23–25]. Relationships are only considered as an outcome in these studies. When looking at the model of Scholl, the clinician-patient relationship is indeed a principle for patient-centredness [8]. In addition, we suggest that a valuable student-patient relationship provides a context which fosters learning mechanisms contributing to patient-centredness as well. Thus, we do not see a relationship solely as an outcome, but also as an important enabler for the development of patient-centredness. Our data supports this since fewer indications for learning were seen when students described their relationship with patients as less meaningful. Similar findings are described in a study by Towle et al. about a longitudinal program, where students of different disciplines learn together with a patient. This study described that building relationships encouraged a deeper understanding of the patients’ lived experiences [26].

When students built meaningful relationships with their patients, engagement occurred, which in turn provided opportunities for the development of patient-centredness - engagement being a learning mechanism [16]. On the other hand, our students struggled with their non-clinical role and how to balance professional and personal proximity in the patient panel. Indeed, concerns have been raised about unhealthy boundaries in student-patient relationships, where students get too involved emotionally [17]. [18] At the same time, educating students in developing these professional boundaries is recognized to be important [17]. [19] [20] We argue that these types of learning interventions, where students are confronted with balancing distance and proximity, help students develop their own professional boundaries. This supports their growth as professionals.

Meaningful relationships between patients and students triggered reflection. This might in part be due to the supportive and non-judgmental environment; an environment that is known to promote students’ reflective processes [27]. Besides, the repeated encounters with patients gave students the opportunity, through multiple reflective cycles, to develop, adjust and test their new understanding and thereby increase the depth of their understanding [21]. [27]

Our data show that the out-of-hospital setting contributes to a relationship between students and patients which supports learning. The importance of this relationship might be better understood by looking at situated learning theory. In this theory, learning is considered to be a complex interchange among the student, teacher and the context in which learning takes place [28]. When considering the students’ educational environment as a community of practice, traditionally legitimate contributors to students’ learning are faculty staff and health care providers. Faculty staff and doctors are seen as the experts, who help students make meaning of their experiences. In these settings, patients are not considered active contributors to students’ learning. Rather, they are seen as passive participants, that are used as objects to teach students [23]. By placing students together with patients in an out-of-hospital setting, a shift in roles and legitimacy seems to occur within the medical education community of practice. Students met patients in their home environment. This setting may strengthen patients in their role of active participants. Instead of being cared for, they now can help students make meaning of what they experience. On the other hand, students do not need to care for patients; their job is to listen and be a learner. The active contribution of the patients to students’ learning is suddenly considered important and legitimate. In the context of a relationship where students feel that they can learn from and with patients, learning mechanisms are triggered.

A change in role and legitimacy due to a different learning environment was also seen in other studies. Towle et al. studied the ‘Health Mentors programme’ where students learn in a non-clinical environment as well. In this program, patients were the mentor of the students. Students identified the mentors’ expertise as a unique feature of this kind of learning, indicating that students view the patients’ contribution to their learning as legitimate [26]. Furthermore, Hendriksen et al. found that physiotherapy and occupational therapy students felt more like learners in a non-clinical environment when practising joint examination on real patients, compared to the clinical environment. This made them accept patients as their teachers from whom they could learn instead of seeing them as patients whom they should care for [24]. However, a similar study with medical students showed that students were sceptical regarding the credibility of the patients’ contribution. Students rated the education as valuable regarding experiential knowledge, but questioned the credibility of patients’ biomedical knowledge and therefore questioned the overall legitimacy of patient-led teaching [25]. In this course, students might have been more focused on the procedure of standard physical examination, rather than patient-centred aspects of the examination. Furthermore, within this course there was a combination of patient-teaching and faculty-led teaching. Perhaps this made students compare between faculty and patients and therefore question the legitimacy of patient-led teaching [25]. Overall, it seems likely that the learning environment influences how students accept patients as active contributors to their learning. In our study, the out-of-hospital setting contributed to a student-patient relationship supportive for learning.

Although the out-of-hospital setting helps in the development of a learning-relationship between students and patients, it is not a prerequisite. For instance, in rural LICs students also reported that they learned from patients, rather than about them [29]. [30] Moreover, Manninen et al. reported that a mutual relationship which enables active patient participation in nursing students’ learning was seen in the hospital setting [31]. Further research should evaluate which elements contribute to a relationship fruitful for learning between students and patients in various settings.

### Implications for further research

The existence of a meaningful relationship was not related to individual students but related to the individual patient contacts students had. All students had meaningful relationships, but not with all of their patients. This was influenced by both patient and student characteristics. In the literature concerns have been expressed that only highly educated patients with similar cultural backgrounds of the students participate in medical education [32]. We found that the lack of common ground counteracted in building meaningful relationships. However, in order for students to serve the wide variety of patients they will encounter, they should learn to build meaningful relationships with diverse patients. Future research should focus on how student and patient characteristics influence the development of a meaningful relationship which fosters learning. Moreover, future research should focus on how students can build relationships with a diversity of patients, including patients with whom they seemingly do not have common ground.

### Strengths and limitations

An important strength of our study was using the realist approach. The CIMO configurations provided insight in distinct aspects of the patient panel design that led to a change in context, which in turn made learning mechanisms occur. These insights can be used by educators when designing or improving interventions targeting patient-centredness. Furthermore, our findings are explained by educational theory which provides insight into *why* the educational intervention works. Other strengths are the open interview strategy without revealing too much of our focus on patient-centredness to the students, minimising socially desirable answers. Besides, students were not directly asked about what they learned, under the assumption that respondents cannot answer such questions reliably [8]. The research student was a fifth-year student, unknown to the third year students. Since the interviewer was the same age, the participants might have had a more open attitude. Lastly, coding and analysis of the interviews was done by two researchers.

A limitation of our study is that it was performed with a convenience sample of students who participated in the patient panel program. Therefore, our results may not be fully transferable to other student populations or other longitudinal programs. Furthermore, our study was not designed to evaluate the extent to which the patient panel improved students’ patient-centeredness. Nonetheless, the goal of this study was to elucidate possible ways in which students’ learning was triggered through a specific context and intervention. We focused primarily on learning processes related to patient-centredness [16]. Thus, this study contributes to the understanding of how a longitudinal and out-of-hospital setting changes the learning context and thereby contributes to patient-centredness. In doing so, it provides a platform for further research into other student populations and settings to examine if these relations will be confirmed.

## Conclusion

The importance of patient-centredness is supported worldwide. This study broadens the understanding of how learning mechanisms, which potentially lead to patient-centredness, are triggered. A longitudinal setting and non-medical role change the context in which students learn. In the context of a meaningful relationship and continuity, learning mechanisms are triggered. The most important learning mechanisms found in this study were: reflecting, contextualizing disease in life stories, broadening perspective and engagement with the patients. We advise faculty to implement educational programs which promote a context of meaningful student-patient relationships and continuity. This can be achieved when students meet patients in a non-clinical role in an out-of-hospital setting for a prolonged period.

## Supplementary information

**Additional file 1 **Supplementary material file- Fostering patient-centredness by following patients *outside the clinical setting an interview study – 20,190,920*. appendix 1, interview guide

## Data Availability

The datasets generated and analyzed during the current study are not publicly available due to restrictions in the ethical consent obtained.

## Abbreviations

CIMO: Context - Intervention - Mechanism – Outcome
GP: General practitioner
LIC: Longitudinal Integrated Clerkship
NVMO-ERB: Ethical Review Committee for the Dutch Association of Medical Education
