# Supplementary material for: Fostering patient-centredness by following patients outside the clinical setting: an interview study
Source: BMC Med Educ. 2020 Jan 15;20:16. doi: 10.1186/s12909-020-1928-9 (PMC6963999; doi:10.1186/s12909-020-1928-9)
Supplement: Supplementary file 1 — Additional file 1 Supplementary material file- Fostering patient-centredness by following patients outside the clinical setting an interview study – 20,190,920. appendix 1, interview guide [file 12909_2020_1928_MOESM1_ESM.docx]

## Appendix 1: Semi-structured interview guide

| **Topics** | **Aim in this part of the interview** |
| --- | --- |
| Theme 1: description patient and context | To let the student describe the patient, the patient’s life, the people involved in the lives and disease of the patient. The effect on the student of following the patient for a longer time. To see if the descriptions are rich or superficial. |
| Theme 2: coping strategy of the patient | To find out if the student can tell something about the coping strategy of the patient, the perspective of the patient. The ability of the student to empathize with the patient. |
| Theme 3: conversations with the patient | To find out to what extent there was conversation and what the content of the conversation was. If and how the student remembers the conversation. Whether the student reflected on these conversations later on. |
| Theme 4: communication and relation with caregivers | To find out what the students notices about the communication and relation with caregivers and whether the students sees the effect this has on the patient. To let them talk about which behaviour they would pursue and which not, and why. |
| Theme 5: shared decision making | To find out if the students notices whether there is shared decision making or not, and the effect of this on the patient. How they notice this. |
